# Susceptibility of Goldfish to Cyprinid Herpesvirus 2 (CyHV-2) SH01 Isolated from Cultured Crucian Carp

**DOI:** 10.3390/v13091761

**Published:** 2021-09-03

**Authors:** Jinxuan Wen, Yao Xu, Meizhen Su, Liqun Lu, Hao Wang

**Affiliations:** 1 National Pathogen Collection Center for Aquatic Animals, Shanghai Ocean University, Shanghai 201306, China; m190100344@st.shou.edu.cn (J.W.); 1911119@st.shou.edu.cn (Y.X.); m180100212@st.shou.edu.cn (M.S.); lqlv@shou.edu.cn (L.L.); 2 National Demonstration Center for Experimental Fisheries Science Education, Shanghai Ocean University, Shanghai 201306, China; 3 Key Laboratory of Freshwater Aquatic Genetic Resources, Ministry of Agriculture, Shanghai Ocean University, Shanghai 201306, China; 4 Pilot National Laboratory for Marine Fisheries Science and Technology, Qingdao 266200, China

**Keywords:** CyHV-2, crucian carp, goldfish, sensitivity, pathology, ornamental fish

## Abstract

Cyprinid herpesvirus 2 (CyHV-2), a member of the Alloherpesviridae family belonging to the genus *Cyprinivirus*, is a fatal contagious aquatic pathogen that affects goldfish (*Carassius auratus*) and crucian carp (*Carassius carassius*). Although crucian carp and goldfish belong to the genus *Carassius*, it is unclear whether they are susceptible to the same CyHV-2 isolate. In addition, the origin of the crucian carp-derived CyHV-2 virus isolate remains unclear. CyHV-2 SH01 was isolated during herpesviral hematopoietic necrosis disease (HVHN) outbreaks in crucian carp at a local fish farm near Shanghai. CyHV-2 SH01 was confirmed by PCR and Western blot analysis of kidney, spleen, muscle, and blood tissue from the diseased crucian carp. Moreover, histopathological and ultra-pathological analyses revealed pathological changes characteristic of CyHV-2 SH01 infection in the tissues of the diseased crucian carp. In the present study, goldfish and crucian carp were challenged with CyHV-2 SH01 to elucidate viral virulence. We found that CyHV-2 SH01 could cause rapid and fatal disease progression in goldfish and crucian carp 24 h post-injection at 28 °C. Experimental infection of goldfish by injection indicated that the average virus titer in the kidney of the goldfish was 10^3.47^ to 10^3.59^ copies/mg. In addition, tissues exhibited the most prominent histopathological changes (cellular wrinkling and shrinkage, cytoplasmic vacuolation, fusion of the gill lamellae, and hepatic congestion) in CyHV-2 SH01-infected goldfish and crucian carp. Thus, crucian carp and goldfish showed a high sensitivity, with typical symptoms, to HVHN disease caused by CyHV-2 SH01.

## 1. Introduction

The order Herpesvirales includes the families *Alloherpesviridae*, *Malacoherpesviridae*, and *Herpesviridae*, which comprise enveloped DNA viruses. Most herpesviruses exhibit long-term persistence (e.g., latency), rather than causing serious disorders in their hosts [1,2,3]. In contrast to other herpesviruses, cyprinid herpesvirus infections could cause high mortality in carp such as common carp (*Cyprinus carpio*), crucian carp (*Carassius carassius*), and goldfish (*Carassius auratus*) [4,5,6,7,8,9,10]. Four species, namely anguillid herpesvirus 1, cyprinid herpesvirus 1, cyprinid herpesvirus 2 (CyHV-2), and cyprinid herpesvirus 3, have been classified into the genus *Cyprinivirus* to date [11]. Of these, CyHV-2 has a narrow host range and is highly virulent; it has spread to the main areas of crucian carp culture worldwide, and especially in China.

Goldfish are cultured as an ornamental pet [12] and were derived from the Prussian carp by artificial selection. The history of goldfish domestication and artificial breeding can be traced back thousands of years in Asia [13], and numerous morphologies and variants have been domesticated as ornamental fish to date [14]. Compared to wild and cultured crucian carp species, ornamental goldfish species have dysmorphic features such as a large head and abnormal fins, which could significantly impact their health and resistance to diseases [15].

Crucian carp is one of the most important freshwater fish species of the *Cyprinidae* family in China. Since the successful application of artificial propagation, especially of all-female Prussian carp (*Carassius gibelio*), which were produced by heterologous sperm gynogenesis to activate embryo development, the annual production of crucian carp around the world has reached about 2772.3 million tons (FAO, 2018).

CyHV-2 infection was initially identified as herpesviral hematopoietic necrosis (HVHN) in goldfish with high mortality in Japan in 1992 [16]. Since then, the virus has spread into new areas, including the United States, Australia, and the United Kingdom [17,18]. CyHV-2 has now been recorded in goldfish populations all over the world. Around the year 2010, CyHV-2 infection in the silver crucian carp (*Carassius auratus gibelio*) instead of the usual host of the goldfish was reported for the very first time [19]. Subsequently, the disease in crucian carp has spread rapidly in many areas, including Hungary, Czechia, Italy, and China [19,20,21,22]. To date, CyHV-2 infection was responsible for considerable economic losses in crucian carp farming, especially in China. It was therefore hypothesized that CyHV-2 pathogenesis may have originated in goldfish. In this study, we determined whether goldfish were susceptible to CyHV-2 SH01 isolated from crucian carp.

## 2. Materials and Methods

### 2.1. Sample Collection and Clinical Examination

Moribund crucian carp (13–15 cm in length) were collected during disease outbreak at a fish farm near Shanghai, China. Dead crucian carp were immediately transported to the laboratory (Shanghai Ocean University). Upon the arrival of each fish, its clinical symptoms were recorded, and then all fish underwent a wet-mount examination to identify the presence of parasites and possibly other microorganisms. For parasites, smears of skin mucous and pieces of gill tissue were carried out by the routine microscopic examination according to a previous protocol [7]. For primary bacterial isolation, the samples obtained from the gill, ascites, heart, liver, and kidney were streaked on freshly prepared Luria–Bertani (LB) medium (Solarbio, Beijing, China), and each was then cultured at 25 °C for about 24 h.

### 2.2. Polymerase Chain Reaction (PCR), Sequence Analysis, and Western Blot

PCR analysis of CyHV-2 and CyHV-3 (KHV) was performed according to the Chinese quarantine standards “Detection method of goldfish hematopoietic necrosis virus (GB/T36194)”and “Detection method of KHV (SN/1674-2014)”. Primer pair sequences were as follows: CyHV-2Hel-F: 5ʹ-GGACTTGCGAAGAGTTTGATTTCTAC-3ʹ, CyHV-2Hel-R:5ʹ-CCATAGTCACCATCGTCTCATC-3ʹ, KHV TK-F: 5ʹ-GGGTTACCTGTACGAG-3ʹ, KHV TK-R: 5ʹ-CACCCAGTAGATTATGC-3ʹ. DNA was isolated using a TIANamp Genomic DNA kit (DP304, Tiangen, Beijing, China) according to the manufacturer’s instructions. PCR amplification was performed using the PrimeSTAR^®^ Max System (R045Q, TaKaRa, Beijing, China). After sequencing (Sangon Biotech Shanghai Co., Ltd. Shanghai, China), the results were bioinformatically analyzed using GenBank blast search. The phylogenic tree was constructed using the DNAMAN version 6 software (Lynnon Corporation, Quebec city, QC, Canada) and MEGA 5.0 software (http://www.megasoftware.net/download_form (28 March 2020)). For Western blot analysis, tissue samples were lysed with RIPA (Beyotime, Shanghai, China) containing a protease inhibitor cocktail. Western blot was performed as previously described [5] using anti-ORF121 polyclonal antibody [4], and the secondary antibody used was goat anti-mouse IgG (H + L) -HRP (BS12478, Bioworld, Nanjing, China).

### 2.3. Light and Electron Microscopy

Histological examinations were performed as described previously [23]; tissues from the fish were dissected, fixed in neutral buffered formalin for 24 h, washed with Dulbecco’s phosphate-buffered saline (DPBS, Sigma, New York, NY, USA), dehydrated with 30% sucrose/DPBS, and embedded in optimum cutting temperature compound (OCT). Eight micrometer sections of all tissue specimens were made using a cryostat (CM1950, Leica, Nussloch, Germany). Sections were stained with hematoxylin and eosin (Sigma, New York, NY, USA) and visualized using a IX71 Olympus microscope coupled to a DP70 Olympus digital camera (Olympus, Tokyo, Japan). Ultrastructural analysis was carried out as previously described [6]. Transmission electron microscopy (TEM) was performed using a transmission electron microscope (FEI Tecnai Spirit).

### 2.4. Virus Amplification and Purification

Virus amplification was performed in crucian carp as described by Xu et al. [24] and Luo et al. [25]. Internal organs were collected and homogenized with a tissue homogenizer under a vented hood. Subcellular fractionation was performed by differential centrifugation with rotation speeds of 3000, 4500, 6000, 8000, 12,000, and 14,800 rpm. After centrifugation, the supernatant was collected for subsequent centrifugation, and the last supernatant was collected with a disposable needle filter with a pore size of 0.22 µm to obtain a purified virus tissue grinding filter solution. The pelleted viruses were dissolved with TN buffer, and aliquots were stored at –80 °C. Additionally, complete genome sequencing of CyHV-2 SH01 was performed using next generation sequencing by the NextOmics company (Wuhan, China). Virus titer values were determined using an established protocol [26]. DNA extracted from purified CyHV-2 particles was sequenced, and CyHV-2 SH01 genome sequencing was commercially performed by the NextOmics company (Wuhan, China).

### 2.5. Fish and Experimental Infection

In order to determine whether the same CyHV-2 SH01 virus could cause fewer or different symptoms in crucian carp and goldfish, we injected crucian carp and goldfish with CyHV-2 SH01 by intraperitoneal injection. Healthy goldfish (length: 12–13 cm) and crucian carp (length: 13–15 cm) were obtained from the Shanghai Ocean University fish breeding farm and acclimated for a week in the laboratory. We randomly divided the fish into the following 4 groups, with 10 individuals in each group: Group A: goldfish injected intraperitoneally with 400 µL virus stock with 10^6^ copies/µL of CyHV-2. Group B: crucian carp were injected intraperitoneally with 400 µL of 10^6^ copies/µL. Group C and D: goldfish and crucian carp, respectively, injected intraperitoneally with 400 µL PBS as control group. The viral DNA copy numbers in the kidneys of the challenged goldfish and crucian carp were measured by real-time PCR 24 h after viral infection. All experimental fish were reared in a tank at 28 °C with a flow-through water system and fed once a day with commercially produced fish food. Deceased fish were tested by CyHV-2 PCR.

### 2.6. Ethics Statement

All animal experiments were performed in complete compliance with the guidelines of the Ethics Review Committee at Shanghai Ocean University.

### 2.7. Quantitative Real-Time PCR Analysis of CyHV-2

Viral DNA was extracted with a DNA Kit from Tiangen (DP304, Tiangen, Beijing, China) as described above. Primers used for real-time PCR were obtained from our previous research (F: 5ʹ-GGACATCAAATCGGCAGCTC-3ʹ, R: 5ʹ-CTCCTCCATGGTCACATCGG-3ʹ) [4]. The reaction mixture (25 μL) contained 12.5 μL TB Green^®^ Premix Ex Taq II (RR820A, TaKaRa, Beijing, China), forward primers and reverse primers at 1 μL each, and cDNA at 100 ng, with nuclease free water at 25 μL. The reaction conditions were 95 °C for 30 s, 95 °C for 5 s, and 60 °C for 30 s for 39 cycles.

### 2.8. Statistical Analysis

All the data obtained were tested for homogeneity of variance before further analysis. All qualitative data were sorted in MS Excel 2016 and analyzed using SPSS software (SPSS standard, version 18.0; SPSS, Inc.). Data analysis was performed using a two-tailed *t*-test, and *p*-values <0.05 or <0.01 were considered statistically significant.

## 3. Results

### 3.1. CyHV-2 Detection in Diseased Crucian Carp and CyHV-2 SH01 Isolation

**Figure 1:**
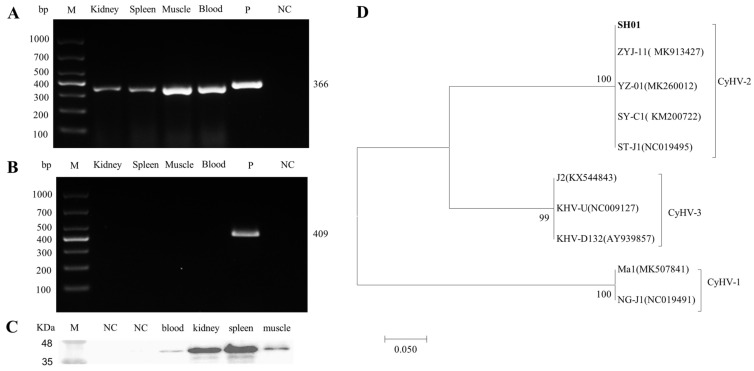
Detection of CyHV-2 in diseased crucian carp and phylogenetic tree construction. (**A**) CyHV-2 PCR analysis of tissue from diseased crucian carp. (**B**) KHV PCR analysis of tissue from diseased crucian carp. (**C**) Western blot analysis of CyHV-2 ORF121 in tissues from the diseased crucian carp. (**D**) The phylogenetic tree was constructed based on amino acids sequences of *helicase*, by using the neighbor-joining method in the MEGA 5.0 software. Bootstrap values of 1000 replications are shown at the nodes. GenBank accession numbers were as follows: CyHV-2(SH01, Bankit2436221; ZYJ-11, MK913427; YZ-01, MK260012; SY-C1, KM200722; ST-J1, NC019495), CyHV-3(J2, KX544843; KHV-U, NC009127; KHV-D132, AY939857), CyHV-1(Ma1, MK507841; NG-J1, NC019491).

Tissue samples from the kidney, spleen, muscle, and blood of diseased crucian carp were assessed using PCR and Western blot. As shown in Figure 1A,B, the target PCR amplification yielded the expected product size at 366 bp, and the Western blot highlighted an expected target protein product size at 40 kDa, confirming the replication of CyHV-2 in the crucian carp tissues analyzed. Compared to the other tissues tested, the kidney and spleen showed a significantly higher viral load, confirming a previous report [9]. A phylogenetic tree was constructed based on the sequences derived from helicase gene PCR products. It showed the clear clustering of the newly determined sequences with previously described CyHV-2 sequences. The isolate was named CyHV-2 SH01 (Figure 1C). The complete genome sequence of CyHV-2 SH01 was deposited in the GenBank database under the accession number Bankit2436221.

### 3.2. Histopathological and Ultrastructural Examination of Tissues from the Diseased Crucian Carp

**Figure 2:**
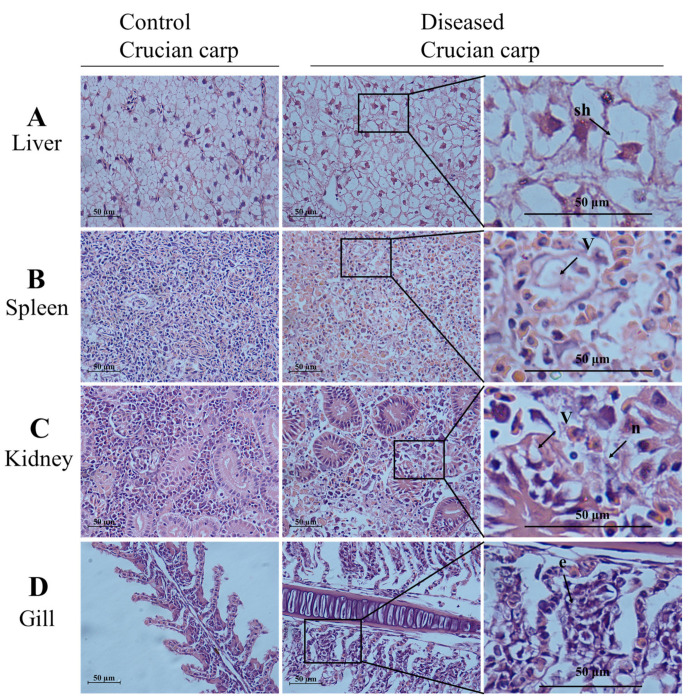
Histological differences in the liver, spleen, kidney, and gill of the diseased crucian carp and control fish (healthy fish). Samples of liver, spleen, kidney, and gill from the diseased fish and the control fish (healthy fish) were fixed, embedded, sectioned, and stained with hematoxylin and eosin (HE). The slides were examined with light microscopy. Scale bars = 50 µm. (**A**) Swelling of hepatocytes (sh) marked by an arrow; (**B**) the arrow shows vacuolation (v); (**C**) focal necrotic lesions (n) and vacuolation (v) were present on the kidney of the diseased crucian carp; (**D**) the arrow shows the exfoliation of epithelial cells (e).

Histopathological changes in tissue samples from diseased and healthy crucian carps were examined, and representative images are shown in Figure 2. In contrast to the control group, semi-thin sections from diseased crucian carp showed cell shrinkage, partial necrosis, and extensive inflammatory cell infiltration (Figure 2). Compared with the control group, inflammatory cell infiltration, cell edema, and steatosis were observed in hepatocytes of the diseased fish (Figure 2A). As shown in Figure 2B, vacuolation was observed in the spleen of the diseased crucian carp. In controls, the kidneys showed no histopathological damage, whereas in infected fish, the kidneys showed extensive histopathological changes including necrosis, nuclear debris, and marginal chromatin (Figure 2C). The gills of the diseased fish exhibited an extensive fusion of the adjacent lamellae and clear collapse and necrosis (Figure 2D). Thus, the diseased crucian carp exhibited symptoms characteristic of goldfish haematopoietic necrosis virus disease [6,23,27].

**Figure 3:**
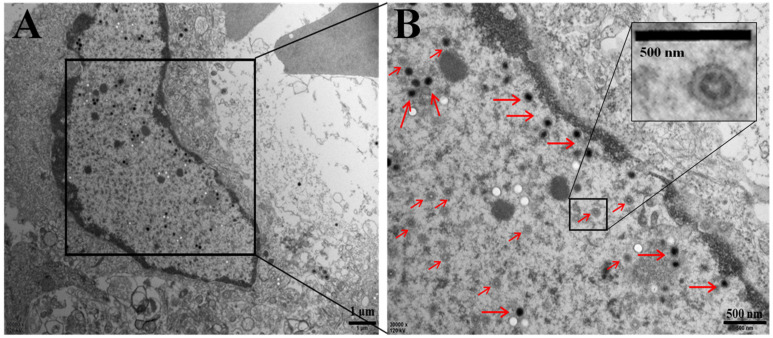
Electron micrograph showing mature virions (red arrow) in a naturally infected kidney cell of crucian carp. (**A**) Scale bar = 1 μm. Insert (**B**): higher magnification of enveloped capsids. Scale bar = 500 nm.

Transmission electron microscopy images of kidney cells from naturally diseased crucian carp (Figure 3) showed numerous 150 to 250 nm spherical virions present across the entire cell. An electron-dense core and an electron-lucent peripheral halo were observed in the enveloped virions (Figure 3B). The complete viral particles appeared to contain a circular structure and the inner ring (Figure 3B). The morphology of these viral particles was consistent with that of CyHV-2 reported previously [22,24].

### 3.3. Gross Signs of Experimental Fish

**Figure 4:**
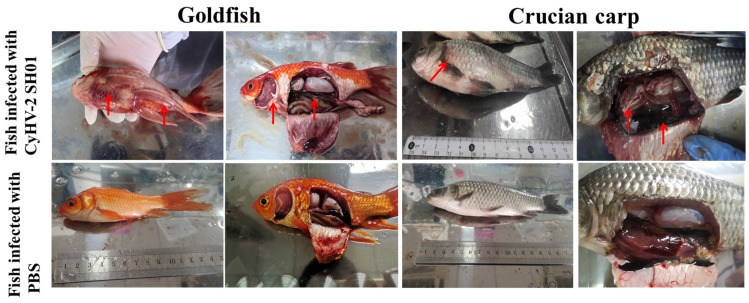
Gross pathology of CyHV-2 in infected goldfish and crucian carp. Goldfish infected by intraperitoneal injection: dorsal and caudal fins bleeding from the base, and liver and spleen enlargement. Crucian carp infected by intraperitoneal injection: gill bleeding, massive abdominal hemorrhage, abdominal swelling and congestion, eyeball protrusion. Red arrows show the representative symptoms of CyHV-2 infection in experimental fish.

As shown in Figure 4, there were no significant differences between the two species after infection. Gross signs of infection in crucian carp and goldfish after viral injection included gill bleeding, body swelling, abdominal hemorrhages, and generalized edema (Figure 4). Compared with crucian carp, goldfish suffered more severe hemorrhage (Figure 4). These symptoms were consistent with those previously reported in fish infected with CyHV-2 [6,22,27,28,29].

### 3.4. Virulence of CyHV-2 SH01

**Figure 5:**
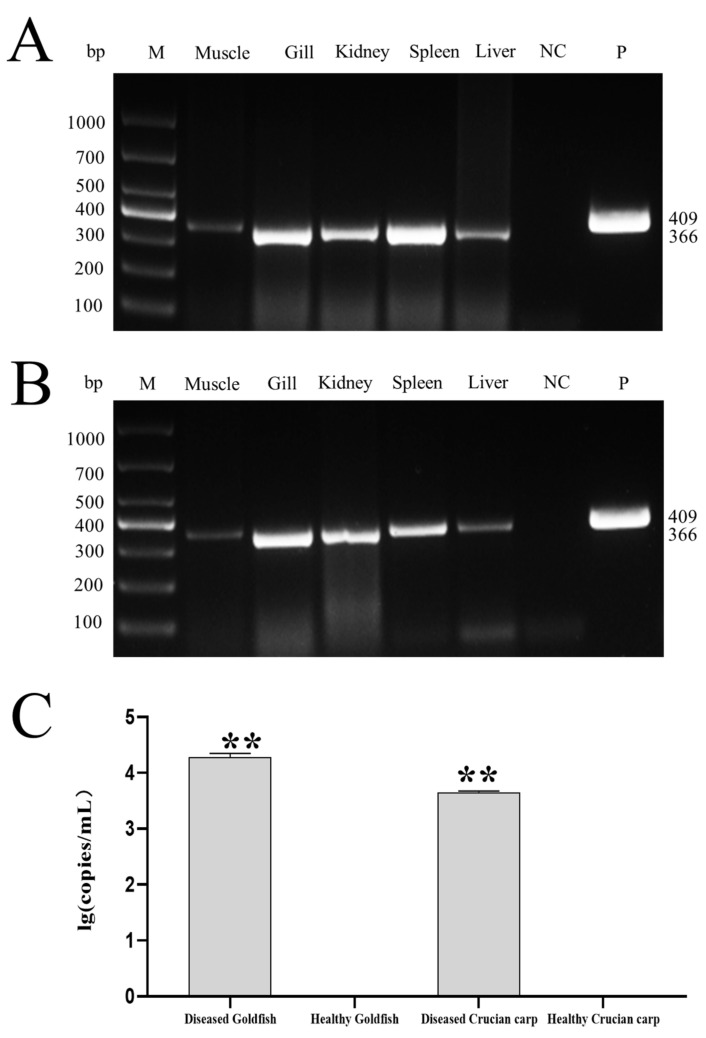
Virulence of CyHV-2 in goldfish and crucian carp. (**A**) CyHV-2 PCR assay of the tissue samples from the experimental goldfish. (**B**) CyHV-2 PCR assay of the tissue samples from the experimental crucian carp (**C**) CyHV-2 DNA load in kidney of experimental goldfish and crucian carp. Data are from at least three independent experiments; error bars represent the standard errors of the means (SEM). Asterisks indicate significant differences relative to the healthy goldfish or healthy crucian carp (ANOVA and Dunnett´s multiple comparison test; ** *p* < 0.01).

**Figure 6:**
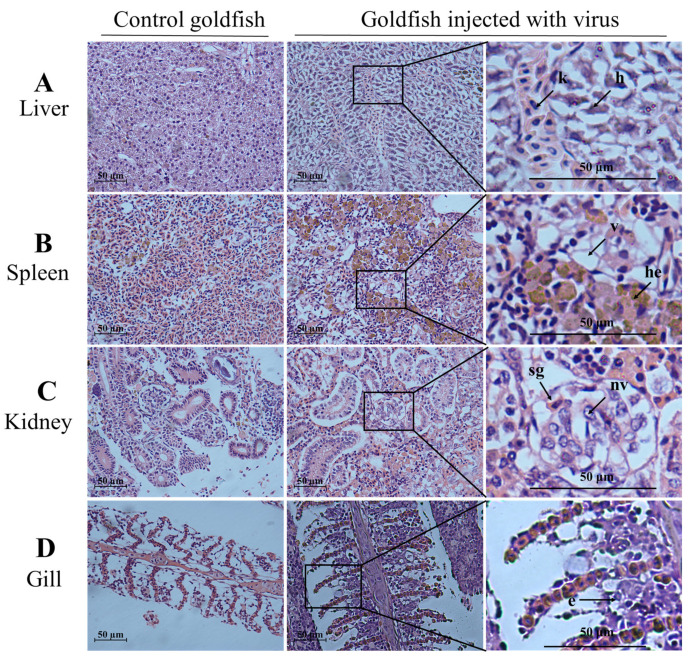
Histological changes in the liver, spleen, kidney, and gill from challenged goldfish (HE staining). Scale bars = 50 µm. (**A**) Extensive necrosis of liver parenchyma. Karyopyknosis (k) and hyperemia (h) marked by an arrow; (**B**) vacuolation of cytoplasm (v) and hemosiderosis (he) in spleen; (**C**) the arrow shows the swelling of glomerular (sg) and nuclear vacuole (nv); (**D**) exfoliation of epithelial cells (e) marked by an arrow.

**Figure 7:**
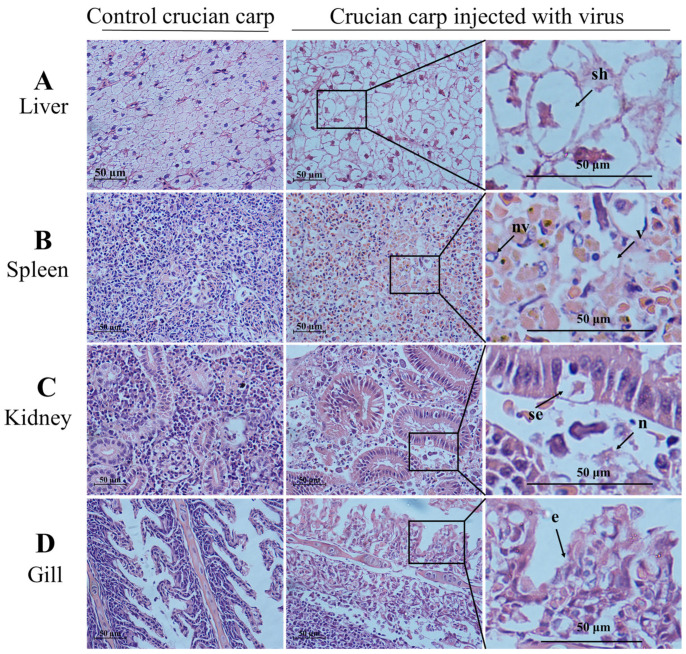
Histological changes in the liver, spleen, kidney, and gill from challenged crucian carp (HE staining). Scale bars = 50 µm. (**A**) swelling of hepatocytes (sh) and decrease of hepatic sinusoid marked by an arrow; (**B**) nuclear vacuole (nv) and vacuolation (v) in spleen; (**C**) the arrow shows the swelling of renal tubular epithelial cell (se) and necrosis (n); (**D**) exfoliation of epithelial cells (e) marked by an arrow.

As shown in Figure 5A,B, CyHV-2 SH01 could be detected in the tested tissues from the goldfish and crucian carp, including muscle, gill, kidney, spleen, and liver. As shown in Figure 5C, the CyHV-2 virus load in goldfish and crucian carp after infection by intraperitoneal injection was 10^3.55^ and 10^2.92^ copies/mg, respectively. This dose of virus caused 100% mortality in goldfish and crucian carp within 24 h post injection with the CyHV-2 SH01. Histological observation of the liver, spleen, kidney, and gill from the virus-infected goldfish and crucian carp showed characteristic pathological changes compared to those from healthy fish (Figure 6 and Figure 7), and karyopyknosis, hyperemia, and swelling of hepatocytes were observed in the liver after CyHV-2 SH01 24 h post-infection (Figure 6A and Figure 7A). The widespread vascular necrosis of hepatocytes was found in both goldfish and crucian carp. In the spleen, clear nuclear chromatin condensation and margination were observed (Figure 6B and Figure 7B). Figure 6C and Figure 7C indicate the extensive necrosis observed in kidneys of the infected goldfish and crucian carp; severe swelling in the renal glomerulus and an increase in melanomacrophage centers were also observed (Figure 7C). In the gill, diffuse hypertrophy and hyperplasia of the branchial secondary lamellae epithelium were observed, as shown in Figure 6D, and gill hyperplasia and the collapse and fusion of the secondary gill lamellae occurred in the gills of infected fish. Consistent with previous reports, characteristic symptoms including extensive necrosis and cytoplasmic inclusions in the kidney, spleen, gill, and liver were observed in the infected goldfish and crucian carp [6,20,22,27,30]. Thus, CyHV-2 SH01 infected goldfish and induced fatal disease with typical symptoms of HVHN, similar to those observed in crucian carp.

## 4. Discussion

CyHV-2 has been circulating widely in crucian carp and goldfish, causing HVHN disease and a marked reduction in crucian carp production in China over the last decade [7,27]. CyHV-2 was first reported in ornamental goldfish and not crucian carp [16], and the source of CyHV-2’s introduction into crucian carp remains unknown. Research in the past decade has focused mainly on virus detection technologies and viral genome sequencing. To date, seven CyHV-2 isolates have been cultivated and described, and their genomes have been sequenced (SY-C1(Genbank accession NO. KM200722), SY(KT387800), ST-J1(NC_019495), 1301(KU199244), CNDF-TB2015(MN201961), YZ-01(MK260012), and YC-01(MN593216)). Of these viral isolates, isolate ST-J1 was isolated from goldfish, and the remaining six isolates were isolated from crucian carp. The available data indicate that there are no significant differences between the CyHV-2 isolates from goldfish and crucian carp [31,32]. Moreover, the full-length genome of SY-C1 from crucian carp shares 98.8% homology with that of ST-J1, which originated in goldfish [31]. Thus, it is suspected that the same CyHV-2 isolate could infect crucian carps and goldfish.

CyHV-2 isolate SH01, which is associated with acute gill hemorrhages and high mortality, was isolated from cultured crucian carp near Shanghai, China in 2019. A PCR assay showed the presence of CyHV-2 SH01 in various tissues including the kidney, spleen, muscle, and blood (Figure 1A). As shown in Figure 1B, the results of a Western blot assay with anti-ORF121 polyclonal antibody were consistent with those of the PCR assay (Figure 2A). Furthermore, the histopathological features observed in the diseased crucian carp from the fish farm were consistent with those described in previous reports (Figure 2) [6,7].

Our experimental infection studies showed similar clinical symptoms in crucian carp and goldfish. External signs in the moribund fish infected with CyHV-2 SH01 were gill pallor and bleeding in the whole body (Figure 3). Additionally, the diseased fish showed signs of hyperemia, bleeding, or coagulation phenomena in the kidney, liver, and spleen (Figure 3). These clinical symptoms were consistent with previously reported symptoms of HVHN disease [6,33,34]. In addition, Ito et al. [30] and Wei et al. [9] suggested that one of the routes of spread for CyHV-2 is through the global trade of asymptomatic infected goldfish. This theory supports the hypothesis that the incidence of CyHV-2 in crucian carp likely originated from a goldfish isolate of CyHV-2. We therefore performed experimental infection studies to compare the viral virulence. As expected, the titer of CyHV-2 SH01 in the kidney of goldfish and crucian carp was 10^3.47^ to 10^3.59^ copies/mg after injection infection (Figure 4). This result is in agreement with our previous research [4]. Histological examination indicated that goldfish infected with CyHV-2 SH01 showed characteristic pathological changes in the kidney, spleen, gills, and liver tissue consistent with those observed in crucian carp (Figure 6 and Figure 7). Thus, highly contagious CyHV-2 isolates are more likely to circulate between goldfish and crucian carp.

In this study, we demonstrated that goldfish show extremely high sensitivity to CyHV-2 SH01 from crucian carp. Since the immersion or bath challenge mimics the natural route of infection in aquatic conditions, immersion experiments are needed to confirm the observations presented here. Moreover, our previous studies indicated that CyHV-2 is often present during the establishment of ponds, leading to the symptomless infection of crucian carp or goldfish [4]. Similar to other members of the family *Alloherpesviridae* within the order herpesvirales, such as CyHV-3 (KHV), CyHV-2 can remain latent in vitro and can be reactivated under stress conditions [10]. In addition, goldfish trading has become immensely popular around the world [15]. Thus, goldfish transportation without strict quarantine may enable CyHV-2 transmission. Several crucian carp ponds are used to raise goldfish in China [35]. Our results suggest that this cultivation mode is a potential route for CyHV-2 introduction into crucian carp.

## Data Availability

Data is contained within the article.
